# Peptide Based Inhibitors of Protein Binding to the Mitogen-Activated Protein Kinase Docking Groove

**DOI:** 10.3389/fmolb.2021.690429

**Published:** 2021-07-01

**Authors:** Anita Alexa, Orsolya Ember, Ildikó Szabó, Yousef Mo’ath, Ádám L. Póti, Attila Reményi, Zoltán Bánóczi

**Affiliations:** ^1^ Biomolecular Interactions Laboratory, Institute of Organic Chemistry, Research Centre for Natural Sciences, Budapest, Hungary; ^2^ Department of Organic Chemistry, Institute of Chemistry, Eötvös Loránd University, Budapest, Hungary; ^3^ MTA-ELTE Research Group of Peptide Chemistry, Eötvös Loránd Research Network (ELKH), Eötvös L. University, Budapest, Hungary

**Keywords:** mitogen-activated protein kinase, protein–protein interaction, peptide inhibitor, cellular uptake, cell-penetrating peptide

## Abstract

Mitogen-activated protein kinases (MAPK) are important regulatory units in cells and they take part in the regulation of many cellular functions such as cell division, differentiation or apoptosis. All MAPKs have a shallow docking groove that interacts with linear binding motifs of their substrate proteins and their regulatory proteins such as kinases, phosphatases, scaffolds. Inhibition of these protein–protein interactions may reduce or abolish the activity of the targeted kinase. Based on the wide range of their biological activity, this kind of inhibition can be useful in the treatment of many disorders like tumors, inflammation or undesired cell apoptosis. In this study a linear binding motif from the RHDF1 protein—a 15 amino acids long peptide—was selected for optimization to increase its cellular uptake but retaining its low micromolar binding affinity. First, we synthesized an octaarginine conjugate that showed efficient cellular uptake. Next, we set out to reduce the size of this construct. We were able to decrease the length of the original peptide, and to increase its cellular uptake with specific chemical modifications. These new constructs bound better to ERK2 and p38 kinases than the original peptide and they showed markedly increased cellular uptake. The new octaarginine conjugate and one of the minimized bicyclic derivatives could inhibit the phosphorylation of intracellular ERK or p38. However, the modulation of MAPK phosphorylation levels by these cell-penetrating peptides were complex, despite that in biochemical assays they all inhibited MAPK-substrate binding as well as phosphorylation. The optimized peptides depending on the applied concentration caused an expected decrease, but also some unexpected increase in MAPK phosphorylation patterns in the cell. This possibly reflects the complexity of MAPK docking groove mediated protein–protein interactions including bone fide MAPK clients such activator kinases, deactivating phosphatases or regulatory scaffolds. Thus, our findings with optimized cell-penetrating “inhibitory” peptides highlight the opportunities but also the pitfalls of docking peptide based MAPK activity regulation and call for a better quantitative understanding of MAPK mediated protein–protein interactions in cells.

## Introduction

Mitogen-activated protein kinases (MAPK) are key regulators of cellular signaling since they play crucial roles in many cellular processes like cell division and survival, differentiation, gene expression, apoptosis and stress-response (Cargnello and Roux, 2011). They are found in eukaryotic cells and are activated by their upstream kinases (mitogen-activated protein kinase kinases, MAPK kinases or MAPKKs) which were previously activated by mitogen-activated protein kinase kinase kinases, MAPKKKs). These kinases form a multi-tiered kinase cascade in which the specific interactions between the activating kinase and their substrate kinase are very important. The activating MAPKKs bind to the docking groove of the cognate MAPK with a specific short linear motif. These linear docking motifs are composed of 8–20 amino acids and have a loose consensus sequence. They contain basic and hydrophobic amino acids which bind to the Common Docking (CD) and hydrophobic region of the docking groove, respectively with a consensus sequence of Ψ (1–3) × (3–7) Φ ×, where Ψ, Φ and × mark positively charged, hydrophobic or any intervening residues, respectively. The docking grooves of paralogous MAPKs are similar but also distinct in a few key amino acid positions, therefore the consensus sequence of the docking motifs has both common and different features.

There are three main subfamilies of mammalian MAPKs: extracellular signal-regulated kinases (ERKs), p38 mitogen-activated protein kinases (p38s), and c-Jun N-terminal kinases (JNKs) (Pearson et al., 2001). ERKs are activated by mitogen signals, while p38s and JNKs by cellular stress (osmotic stress, heat shock) and inflammatory cytokines. The same surface of the MAPK’s docking groove serves as the binding site for not only the activator kinases but also for their inactivating phosphatases, downstream substrates, and any signaling partners having these motifs. By inhibition of the interaction between MAPKs and their signaling partners we may potentially influence MAPK signaling. These MAPKs play important roles in many pathological events as tumor genesis and progression, metastasis formation and inflammatory processes. Thus, these protein–protein interactions (PPIs) are ideal therapeutic targets, but their inhibition is very challenging. Designing small molecule PPI inhibitors may be more difficult, because of a) the interaction happens *via* a big surface, bigger than in the case of other interactions (e.g., enzyme-substrate, ligand-receptor) (Smith and Gestwicki, 2012) b) these surfaces are commonly flat and there are only few grooves or deep pockets (Hopkins and Groom, 2002), c) there are no small endogenous ligands that could be used as a template for drug design. Although there are several successful small molecule inhibitors (Nero et al., 2014; Lu et al., 2020), larger compounds may be better to inhibit PPI’s. One extensively studied family of this kind of molecules is peptides (Nevola and Giralt, 2015; Tsomaia, 2015). They have several advantages (Craik et al., 2013), and their higher selectivity toward protein targets may result in less side effects. They can be easily modified with unnatural amino acids that increases their biological and chemical diversity. Their metabolism results in amino acids that are non-toxic products. Unfortunately, they have some disadvantages too; lower metabolic stability, cell-permeability and oral availability. Peptides that can bind to a protein have specificity because of their well-defined structure in their protein domains. Unfortunately, short peptides related to these parts of the binding domains lose these specific secondary structures. There are many chemical approaches to avoid this “side-effect” of size reduction of biologically active protein domains (Nevola and Giralt, 2015; Tsomaia, 2015), for example by producing cyclic peptides (Lennard et al., 2019; Sarkar et al., 2020; González-Muñiz et al., 2021), macrocyclic peptides (Dougherty et al., 2017; Krüger et al., 2017; González-Muñiz et al., 2021), stapled peptides (Walensky et al., 2004; Dietrich et al., 2017; Jeganathan et al., 2019), or bicyclic peptides (Trinh et al., 2016). Because the RAS-ERK signaling pathway plays an important role in the development of many tumors, it is a promising target to develop therapeutically active molecules (García-Gómez et al., 2018). Therefore, there has been considerable interest in developing small molecule inhibitors that bind to the MAPK docking groove (Burkhard et al., 2009; Sammons et al., 2019). The first peptide that inhibited the activation of ERK was derived from its activator protein kinase MEK1 (Kelemen et al., 2001). This 12 amino acids long peptide corresponding to the N-terminus of MEK1 inhibited the activation of ERK *in vitro* and its cell-permeable derivative was effective in cells. Further peptides were identified as inhibitors in later studies (Bardwell et al., 2003; Ho et al., 2003; Gaestel and Kracht, 2009). The linear docking motifs that are used by MAPKs to increase their signaling specificity (Tanoue and Nishida, 2002; Reményi et al., 2006) may all be good candidates to develop PPI inhibitors. The structural requirements and the mode of binding of these linear docking motifs was described earlier (Garai et al., 2012) and was used to identify further motifs (Zeke et al., 2015). One of these motifs (its sequence: SLQRKKPPWLKLDIPS) found in the Inactive rhomboid protease 1 (RHDF1) showed strong binding to ERK2 (Zeke et al., 2015). Although these peptides can bind very selectively and sometimes strongly to their target proteins, there are often limitations of their use in cells, due to their lack of cell-permeability in particular. The cellular uptake of these peptides can be increased using cell-penetrating peptides, and these positively charged peptides are known to be able to deliver a wide range of cargos into the cytosol (Hudecz et al., 2005). In former studies, we used these peptides successfully to deliver small molecules (Miklán et al., 2007; Bánóczi et al., 2008b; Szabó et al., 2016) or peptides (Bánóczi et al., 2007; Bánóczi et al., 2008a; Világi et al., 2008) into cells or tissue.

In this study we present the structural optimization of the linear binding motif from RHDF1 which, in turn, led to the development of cell-permeable modulator of MAPK phosphorylation. The synthesis and biological characterization of its cell-penetrating conjugate and modified derivatives are described and our results indicate that the sequence can be reduced and modified to enhance cellular uptake without compromising, or even slightly improving, MAPK binding. These new peptides may internalize efficiently and can inhibit the PPIs between ERK2 or p38 and their substrates, therefore, they may be suitable to modify the ERK or p38 signaling pathways in cells.

## Materials and Methods

All amino acid derivatives, and Rink-amide MBHA resins were purchased from IRIS Biotech GmbH (Marktredwitz, Germany), whereas N,N-diisopropylethylamine (DIEA), 1-hydroxybenzotriazole (HOBt), N,N′-diisopropylcarbodiimide (DIC), trifluoroacetic acid (TFA), 1.8-diazabicyclo[5.4.0]undec-7-ene (DBU), thioanisole, 5(6)-carboxyfluorescein (Cf), triisopropylsilane (TIS) and 1,2-ethanedithiol were FLUKA (Buchs, Switzerland) and Sigma Aldrich (Budapest, Hungary) products. Dabcyl acid [4-((4-(dimethylamino)phenyl)azo)benzoic acid] was ordered from (AAT Bioquest, Inc., Sunnyvale, CA). Solvents for syntheses and purification were obtained from Molar Chemicals Kft (Budapest, Hungary). The buffers were prepared with ion exchanged distilled water.

### Synthesis of Peptides

All peptides were synthesized manually on Rink-amide MBHA resin (0.3 g, 0.69 mmol/g) by Fmoc/^*t*^Bu strategy as was described earlier (Szabó et al., 2016). After the cleavage of the last Fmoc protecting group peptide for conjugation was chloroacetylated on the free *N*-terminal α-amino group using the pentachlorophenyl ester of chloroacetic acid in 5 eqv. on resin for overnight. The Cf and Dabcyl group were attached to the *N*-terminal of peptides too *via* the α-amino group using their acid and HOBt-DIC coupling reagents. When the peptid contained both Cf and Dabcyl a Lys was incorporated into the *C*-terminal and Cf was coupled to its ε-amino group in solution (DMF) using DIC-HOBt coupling reagents in 1.1 eqv. to the peptide. The chloroacetylated peptides were cleaved from the resin with 5 mL TFA containing 0.375 g phenol, 0.25 mL distilled water and 0.25 mL TIS as scavengers. Peptides without chloroacetyl group were cleaved using the same mixture but 0.25 mL thioanisole and 0.125 mL 1,2-ethanedithiol were applied instead of TIS. Crude products were precipitated by dry diethyl-ether, dissolved in 10% acetic acid and freeze-dried. The peptides were purified by RP-HPLC on a semipreparative Phenomenex Jupiter C18 column (250 × 10 mm I.D.) with 10 μm silica (300 Å pore size). Flow rate was 4 mL/min. Linear gradient elution was applied.

Conjugation was carried out in TRIS buffer (0.1 M, pH = 8.2). The cysteine containing peptides were added slowly to the solution of chloroacetylated peptide. The conjugate was isolated from the reaction mixture by RP-HPLC after lyophilisation.

The cyclic peptides were prepared by the formation of thioether bond between chloroacetyl (on the side chain of lysine) and thiol (in the side chain of cysteine) group. During the peptide synthesis the Lys residue was protected with Dde. After the coupling of Dabcyl group to the *N*-terminal amino group the Dde group was cleaved on the resin by 2% hydrazine hydrate (5 min + 5 min) and the resin was washed with DMF (8 × 0.5 min). After the cleavage chloroacetyl group was attached to the side chain of lysine using 5 eqv. of chloroacetic acid pentachlorophenyl ester on resin and the peptide was cleaved from the resin using TFA cleavage mixture. The cyclic peptide was produced by dissolving the purified peptide precursor (1 mg/mL) in 0.1 M TRIS buffer (pH 8.1) and stirring overnight at room temperature. The reaction solution was then lyophilized, and purified on HPLC.

For the synthesis of bicyclic peptides linear precursor peptides were prepared with three cysteine residues. Then the purified peptides were reacted with thiol reactive linker tris(bromomethyl)benzene (TBMB). The precursor peptides were dissolved in 0.1 M TRIS buffer (pH 8.1), while TBMB (1.5 eqv) was dissolved in acetonitrile. Then the two solutions were combined and stirred overnight, the final concentration of the peptide was 1 mM. The reaction solution was then evaporated to remove acetonitrile and lyophilized. The crude product was purified by RP-HPLC.

The purified compounds were characterized by analytical RP-HPLC and ESI-MS (Table 1 and Supplementary Material).

**TABLE 1:** Characterisation of peptides.

Sequence	Code	R_t_^a^	ESI-MS^b^		K_d_ (µM) ± sd^c^/EC_50_ (μM)^d^	
			M_cal._	M_meas._	ERK2	p38
*H*-SLQRKKPPWLKLDIPSC-*NH*_*2*_	**1**		2007.1	2006.4	**1.8 ± 0.3**	**1.1 ± 0.3**
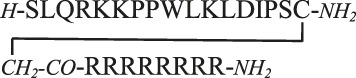	**2**	10.7	3315.0	3315.4	**0.6 ± 0.3**	**n.d.**
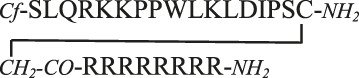	**3**	14.2	3673.4	3673.9	**n.d.**	**n.d.**
*H*-KKPPWLKLDI-*NH*_*2*_	**4**	12.2	1236.5	1236.3	**15.0 ± 1.1**	**n.d.**
*H*-RRPPWLRLDI-*NH*_*2*_	**5**	12.8	1319.8	1319.9	**19.5 ± 2.6**	**10.0 ± 1.1**
*H*-RRPPWLRLDIRR-*NH*_*2*_	**6**	11.4	1633.0	1632.6	**5.2 ± 1.4/12.9^d^**	**n.d./3.7^d^**
*Cf*-RRPPWLRLDIRR-*NH*_*2*_	**7**	12.2	1991.3	1991.2	**n.d.**	**n.d.**
*H*-RRRPPWLRLDIRR-*NH*_*2*_	**8**	10.8	1788.1	1788.1	**1.3 ± 0.1/3.5^d^**	**1.4 ± 0.3/1.3^d^**
*Cf*- RRRPPWLRLDIRR-*NH*_*2*_	**9**	13.3	2146.4	2146.1	**n.d.**	**n.d.**
*Dabcyl*-RRRPPWLRLDIRRK-*NH*_*2*_	**10**	13.3	2167.5	2167.3	**4.6 ± 0.6/8.3^d^**	**1.0 ± 0.4/3.1^d^**
*Dabcyl*-RRRPPWLRLDIRRK(*Cf*)-*NH*_*2*_	**11**	14.7	2525.8	2526.4	**n.d.**	**n.d.**
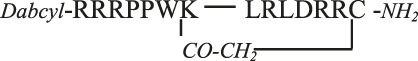	**12**	13.8	2197.2	2197.2	**4.1 ± 0.3**	**1.7 ± 0.4**
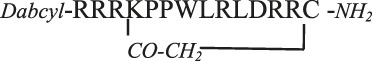	**13**	13.8	2197.2	2198.0	**1.0 ± 0.2**	**1.0 ± 0.7**
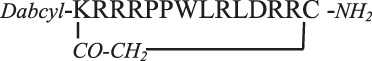	**14**	13.8	2197.2	2198.0	**5.1 ± 0.4**	**2.1 ± 0.6**
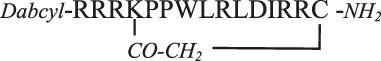	**15**	14.2	2310.3	2310.4	**1.5 ± 0.3**	**1.2 ± 0.6**
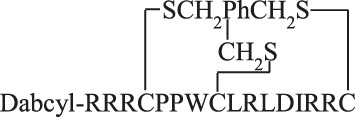	**16**	14.7	2462.3	2462.5	**0.9 ± 0.2**	**1.4 ± 0.4**
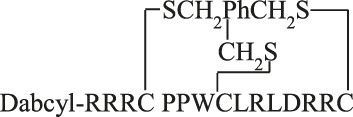	**17**	14.8	2349.2	2349.2	**4.5 ± 0.6**	**3.2 ± 0.7**

^a^Analytical RP-HPLC was done on Nucleosil 120-3 C18 column (4.6 mm × 150 mm, 5 μm, 100 Å). The applied linear gradient elution was 0 min 0% B, 2 min 0% B, 22 min 90% B at 1 mL/min flow rate. The detection was carried on at *λ* = 220 nm.

^b^ESI-MS.

^c^K_d_ was determined by fluorescence polarization assay.

^d^EC_50_ values were determined by an *in vitro* MAPK activity assay, n.d. not determined.

All experiments were carried out at least in 3 independent measurements (Rt, retention time; Cf, carboxafluresceine).

### *In Vitro* Cell Culturing

HL-60 (ATCC^®^ CCL-240™) human promyelocytic leukemia cells were grown in RPMI-1640 supplemented with 10% FCS (l)-glutamine (2 mM) and gentamicin (160 μg/mL). Cells were maintained in plastic tissue culture dishes at 37°C with a humidified atmosphere containing 5% CO_2_/95% air.

HEK-293T (ATCC, CRL-3216) were cultured in Dulbecco’s modified Eagle’s medium (DMEM, Gibco) supplemented with 10% fetal bovine serum (FBS) and gentamicin (50 μg/mL) at 37°C in an atmosphere of 5% CO_2_.

### Cellular Uptake Profile of Compounds by Flow Cytometry and Confocal Microscopy

To study the cellular uptake of fluorescent labeled peptides and conjugate by flow cytometry, 10^5^ HL-60 cells per well were plated on 24-well plates and were incubated for 24 h at 37°C. Then cells were treated with the solution of compounds in serum-free media for 90 min. The cellular uptake was analysed at 1, 5 and 10 µM concentrations. Cells treated with serum-free media were the control. At the end of incubation, treatment solutions were removed, and the cells were treated with 100 μL trypsin for 2 min to remove the membrane proteins in order to eliminate non-specific binding of conjugates. The effect of trypsin was terminated by 900 μL HPMI containing 10% fetal calf serum, and the cell suspensions were transferred into FACS-tubes. After centrifugation at 216*g* at 4°C for 5 min the supernatant was removed. Then cells were resuspended in 500 µl HPMI, and the intracellular fluorescence intensity of HL-60 cells was monitored (on channel FITC LP505; emission at *λ* = 505 nm; LP 505, BP 530/30) by flow cytometry (BD LSR II, BD Bioscience, San Jose, CA, equipped with 488 nm; Coherent Sapphire, 22 mW laser.) which is proportional to the cellular uptake. Data were analysed with FACSDiVa 5.0 software. Statistical analysis was done using two-sided independent Student’s *t*-test.

The cellular uptake of the fluorescent labeled peptide into HEK-293T cells were analyzed by fluorescence microscopy (Cytation 3, BioTek Instruments) and two peptides were further examined by confocal laser scanning microscopy (Zeiss LSM-710 system). Cells were incubated with peptides in serum-free medium (DMEM) for 3 h at 37 °C and then washed twice with 200 μl of serum-free medium before analyzing.

### *In vitro* MAPK Binding and Activity Assays

ERK2 and p38 proteins used for fluorescence polarization (FP) measurements were recombinantly expressed in *Escherichia coli* and purified according to Garai et al. The purified and concentrated proteins were subjected to fluorescence polarization based binding measurements (Garai et al., 2012) using 50 nM Cf-labeled Cf-RHDF (Cf-SLQRKKPPWLKLDIPSC) and the data was analyzed using OriginPro. For direct measurements the concentration of ERK2 and p38 protein was increased, and for competitive measurements the protein-labeled peptide complex at 60–80% complex formation was titrated with increasing concentration of unlabeled peptide and the amount of the protein-labeled peptide complex (fraction bound) was determined using Cytation 3 (BioTek Instruments) plate reader in 384-well black plates. The direct measurement gives the K_d_ between the labeled peptide and the MAPK, while the competitive titration experiment with the unlabeled peptide gives the Ki for the unlabeled peptide competing off the labeled peptide, which is a good proxy for the binding affinity of the unlabeled peptide to the protein. All of the FP measurements were performed in assay buffer: 20 mM Tris (pH 8.0), 100 mM NaCl, 0.05% Brij35, and 2 mM TCEP. Fraction bound of the labeled ligand was calculated based on direct binding and competitive binding equations described in (Roehrl et al., 2004; Simon et al., 2020). The Kd and Ki values were obtained by fitting the FP data, measured in triplicates, to a direct binding equation or to a competitive binding equation using the OriginPro software.

In order to determine the inhibitory effect of RHDF-peptides, we used a protein fragment complementation based assay. MAPK activity was monitored based on the association of two protein constructs containing a phoshorylated substrate reporter sequence including a MAPK docking motif or a phospho-binding domain, where these had an the *N*-terminal or *C*-terminal fragment of the Nanobit Luciferase enzyme as a fusion tag (Promega Nanobit^®^ PPI Starter Systems). Upon MAPK activity, these two proteins bind and form the active luciferase enzyme oxidizing its substrate (coelenterazine) and ultimately giving some luminescent signal. The initial rates of the reactions were calculated and were compared for the applied inhibitor’s concentration, and then EC_50_ values were determined using dose response fitting (OriginPro) (see in Supplementary Figure S26). Reactions were carried out in a buffer of 50 mM HEPES, pH 7.5, 100 mM NaCl, 5 mM MgCl_2_, 0.05% IGEPAL, 50 μM Coelenterazine h, 5% glycerol, 2 mM DTT, 1mM TCEP, 0.5 mg/ml BSA containing 1 nM active ERK2 (pp-ERK2) or p38 (pp-p38α) and were started by the addition of 2 mM ATP. Luminescence was measured using a luminescence plate reader (Cytation 3, BioTek) in white 96-well plates.

### MAPK Phosphorylation Levels in Cells by Western-Blot Analysis

To study the effect of peptides on the ERK pathway EGF stimulation and Western-blot analysis were used. HEK-293T cells were plated on 48-well plate and were serum-starved for overnight. Then cells were treated with the Cf-peptide at 20µM for 5 h in 100 μL DMEM and then stimulated by addition of EGF (Sigma, E9644) in 100 ng/mL concentration. Stimulation was terminated at different time points by adding 35 μL 4× SDS loading buffer. Cells were lysed and 10 μL of each sample was subjected to SDS-PAGE. The proteins of the SDS-gel were transferred to PVDF-membrane by Trans-Blot Turbo Transfer System (Bio-Rad) and the membrane was incubated first by phospho-p44/42 MAPK (ERK1/2) (Thr202/Tyr204) rabbit primery antibody (Cell Signaling, #9101) in 1:2,000 dilution and then with goat anti-rabbit HRP conjugated secondary antibody (Cell Signaling, #7074) in 1:5,000, and after stripping the membrane by anti-p44/42 MAPK (ERK1/2) mouse primary antibody (Cell Signaling, #4696) in 1:2,000 and then with goat anti-mouse HRP conjugated secondary antibody (Merck Milipore, 401215) in 1:10,000. Immobilon^®^ ECL Ultra Western HRP Substrate reagent (Millipor, WBK L S0500) and Fluorchem FC2 gel documentation system (Alpha Innotech) were used for developing the membrane. The intensities of the protein bands were determined by densitometry and monitored after the stimulation in time. Later the effect of the most effective peptide (bicyclic peptide **16**) on the ERK and p38 pathway was examined by Western-blot. In this case HEKT cells were treated with several concentrations of the peptide and then were stimulated by 50 or 200 ng/ml EGF for studying ERK pathway, and 0.4 M Sorbitol for the p38-pathway. Sorbitol induces osmotic stress to the cells which generates activated p38-level (pp-p38), since p38 protein is one of the stress-sensor of the cell. After 10 min cells were lysed and subjected to SDS-PAGE and then subjected to Western-blot as earlier described. Activated (i.e., phosphorylated) ERK and p38 were detected by phospho-p44/42 MAPK (ERK1/2) (see above) and phospho-p38 MAPK (Thr180/Tyr182) rabbit primary antibody (Cell Signaling, #9215) in 1:2000 dilution, respectively. The total ERK and p38 content of the samples were determined by anti-p44/42 MAPK (ERK1/2) mouse primary antibody and anti-p38α MAPK mouse mAb (Cell Signaling, #9228), respectively. Anti-alfa-tubulin mouse mAb antibody (Sigma, T6199) in 1:10,000 was used for loading control. As secondary antibody anti-rabbit antibody (IRDye^®^ 800CW Goat anti-Rabbit IgG, #926-32211) in 1:5,000 and secondary anti-mouse antibody (IRDye^®^ 680CW Goat anti-Mouse IgG, #926-68070 in 1: 10,000 were used. Protein bands were detected and quantified with LI-COR Odyssey^®^ Clx infrared imaging system. Statistical analysis was done using two-sided independent Student’s *t*-test.

## Results

### Design of Inhibitor Peptides

MAPKs are important regulatory proteins with dozens of substrates and they bind their substrates with docking motifs. The S^1^LQRKKPPWLKLDIPS^16^ peptide (**1**) located in the RHDF1 protein is one of these linear binding motifs and can bind to ERK2 and p38 in their docking groove. The bound peptide may inhibit protein–protein interaction and thus blocks the activity of these MAPKs. Our purpose was to develop cell-permeable inhibitors based on the RHDF1 motif. In the first trial a conjugate of the native sequence (**2**) with octaarginine as a cell-penetrating tag was synthesized. Then optimization of the original sequence was carried out to increase intracellular stability and cellular uptake. To reduce the size of the original peptide, several amino acids were eliminated from both termini (**4**). For MAPK binding the Lys^6^, Lys^7^, Leu^10^ and Leu^12^ were found to be important (Zeke et al., 2015). The presence of Ile^14^ as the third interacting site in the hydrophobic groove is not mandatory but may enhance the strength of binding. Thus, in our first truncated construct the flanking regions (“SLQR” on *N*- and “PS” on *C*-terminus) were removed but all necessary amino acids and Ile^14^ were retained in the sequence. As the docking groove prefers both Lys and Arg, all lysine residues were replaced by arginine in the next step (**5**). This modification may increase the cellular uptake of the peptide. For efficient internalization there is a necessary number of Arg residues (Mitchell et al., 2000; Futaki et al., 2001), thus additional arginines were added to both termini to enhance the internalization of the peptide (**6, 8**). Finally, a Dabcyl group as internalization enhancer was attached to the *N*-terminus (**10**). To improve the *in vivo* stability of linear peptides cyclization is often used. However, cyclization may increase the specificity, too. Therefore, cyclic and bicyclic derivatives (**12–17**) were prepared. In some cyclic and bicyclic constructs the Ile^14^ was left out to study its effect on binding. In order to study the cellular uptake fluorescently labeled peptides were synthesized. In these constructs 5(6)-carboxyfluorescein (Cf) was attached to the *N*-terminus or to the ε-amino group of Lys at the *C*-terminus (**3**, **7**, **9** and **11**). All peptides were produced by solid phase peptide synthesis using Fmoc/*t*Bu strategy. Peptides were purified by RP-HPLC and characterized by analytical RP-HPLC and ESI-MS (Table 1 and Supplementary Material).

### Binding of Peptides to MAP Kinases

The binding of peptides was tested with two MAPKs, ERK2 and p38α. Fluorescently labeled RHDF-1 peptide was used as the binding partner of the MAPKs and fluorescence polarization was measured to calculate the binding constant. The binding affinity of peptides were determined in competitive binding assay (K_d_ or K_i_) (Table 1 and Supplementary Figures S24,S25). The conjugation of octaarginine, a cell-penetrating peptide (peptide **2**) slightly increased binding affinity. When the original peptide was shortened (**4**) its binding significantly decreased (8.3 times weaker). The substitution of Lys residues with arginines (**5**) resulted in no change in binding. Extension of the *C*-terminus by two Arg (**6**) enhanced the binding by 3.5 times; addition of one Arg to the *N*-terminus (**8**) further increased binding that became slightly better than that of the original sequence. The K_d_ value of cyclopeptides was dependent on the ring size. The best one was when the cycle contained all amino acids except of the positively charged *N*-terminus (three Arg residues) (**13**). The binding of this construct was one of the best and better than that of the original sequence. . It should be noted that the binding of cyclic peptides with (**15**) or without (**13**) Ile was the same. Based on these results bicyclic peptides with (**16**) or without (**17**) Ile were tested. In these cases the presence of Ile could increase the binding ability and this bicyclic peptide (**16**) showed the strongest binding to ERK2. The peptides bound to p38 similarly albeit with somewhat stronger K_d_ compared to ERK2 (with the exception of **16**).

As peptides could bind to both ERK2 and p38, their inhibitory effect on the activity of these enzymes was measured *in vitro* (Table 1 and see Supplementary Figure S26). The EC_50_ was the lowest in case of the longest peptide that was extended by Arg residues at both termini (peptide **8**). Elimination of an Arg from the *N*-terminus (peptide **6**) reduced the inhibitory effect; its EC_50_ value was 3.7 times higher than that of the longer peptide (**8**). Unfortunately, the attachment of Dabcyl group (peptide **10**) increased the EC_50_ value by 2.4 times. For ERK2, where the EC50 was measured parallel to the K_d_ for three peptides, we found that the best binding peptide had the lowest EC50 value, showing that binding affinity agrees with peptide mediated inhibition for this MAPK.

### Cellular Uptake of Peptides and Conjugates

**FIGURE 1:**
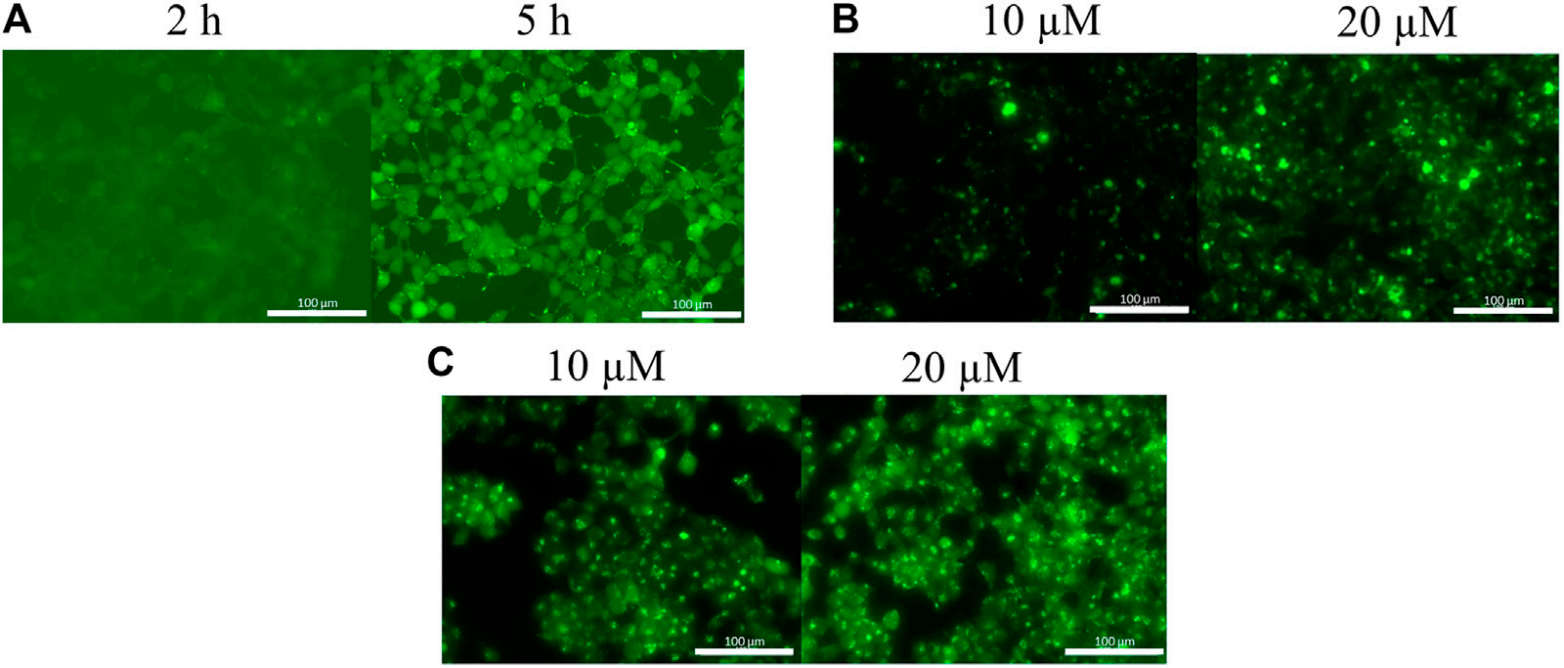
Cellular uptake of peptides into HEK-293T cells. **(A)** *Cf*-RRPPWLRLDIRR-*NH*_*2*_ in 10 µM concentration, **(B)** *Cf*-RRRPPWLRLDIRR-*NH*_*2*_ after 3 h, **(C)** *Dabcyl*-RRRPPWLRLDIRRK(*Cf*)-*NH*_*2*_ after 3 h.

The uptake of the original peptide (**1**) and its shorter derivatives (**4** and **5**) was poor (data is not shown), but it could be enhanced by different modification, for example by additional arginine(s) (**7** and **9**) or a Dabcyl-group (**11**). When arginine residues were incorporated into the peptide at the *C*- (Figure 1A, peptide **7**) or at both termini (Figure 1B, peptide **9**) then time and concentration dependent internalization of peptides was noticeable. Addition of a Dabcyl-group to the *N*-terminus (Figure 1C, peptide **11**) increased the peptide internalization: cells had higher fluorescence intensity even at 10 µM concentration.

**FIGURE 2:**
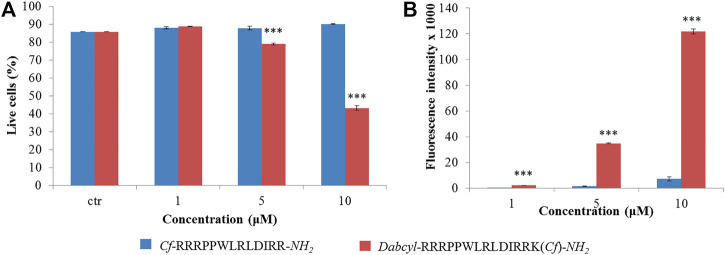
Quantification of the internalization of *Cf*-RRRPPWLRLDIRR-*NH*_*2*_ and *Dabcyl*-RRRPPWLRLDIRRK(*Cf*)-*NH*_*2*_ into HL-60 cells by flow cytometry. The HL-60 cells were treated at different concentrations for 90 min and **(A)** the percent of live cells and **(B)** their average fluorescence intensity was determined by flow cytometry. Error bars show SD calculated based on three independent measurements. Statistical significance between the percent of live cells in the case of control and the peptide treated samples was determined with two-sided independent Student’s *t*-test (***p < 0.001) **(A)**. Statistical significance between the internalization of two peptides was determined with two-sided independent Student’s *t*-test (***p < 0.001) **(B)**.

Because peptide **9** and **11** were the most effective based on fluorescence microscopy images, their internalization was further studied. Flow cytometry measurements were used to assess peptide internalization. HL-60 cells were treated by peptide **9** and **11** in 1, 5 and 10 µM concentrations for 90 min and the fluorescence intensity of cells was measured (Figure 2). The presence of live cells showed that the Dabcyl-containing peptide (**11**) affected cell viability at the two highest concentrations and the number of live cells decreased to 40% at 10 µM (Figure 2A). The fluorescence intensity of the Dabcyl-containing peptide at 1, 5 and 10 µM was 9.2, 23.5 and 16.9 times higher, respectively (Figure 2B).

**FIGURE 3:**
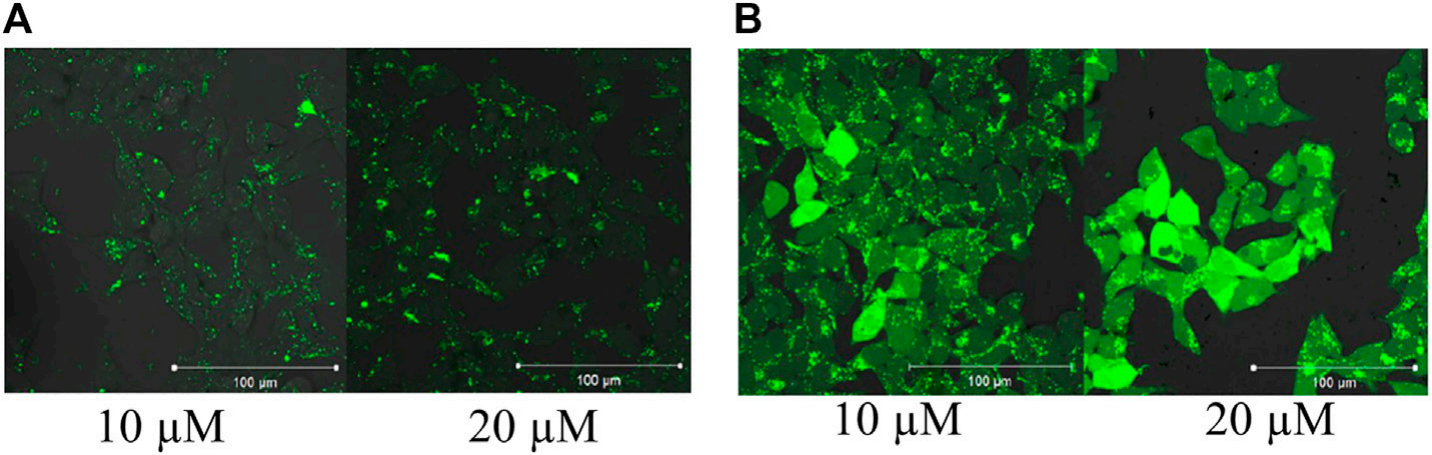
Intracellular distribution of *Cf*-RRRPPWLRLDIRR-*NH*_*2*_ and *Dabcyl*-RRRPPWLRLDIRRK(*Cf*)-*NH*_*2*_ in HEK-293T cells. Cells were treated with 10 and 20 µM peptides for 3 h and the intracellular distribution of peptides was studied by fluorescence confocal microscopy.

When the internalization of the two peptides was confirmed by fluorescence microscopy, their intracellular distribution was examined. HEK-293T cells were treated with the solution of peptide **9** and **11** for 3 h and analyzed by fluorescence confocal microscopy (Figure 3). This analysis showed that the Dabcyl-group affected the peptide internalization pathway. Without the Dabcyl-group the peptide went through the cell membrane only *via* endocytosis since its intracellular distribution was punctuated (Figure 3A). The Dabcyl-group containing peptide showed a more diffuse distribution even at 10 µM which was further increased at 20 µM concentration (Figure 3B).

### Inhibition of Intracellular ERK or p38 by an Octaarginine Conjugate and a Bicyclic Peptide

**FIGURE 4:**
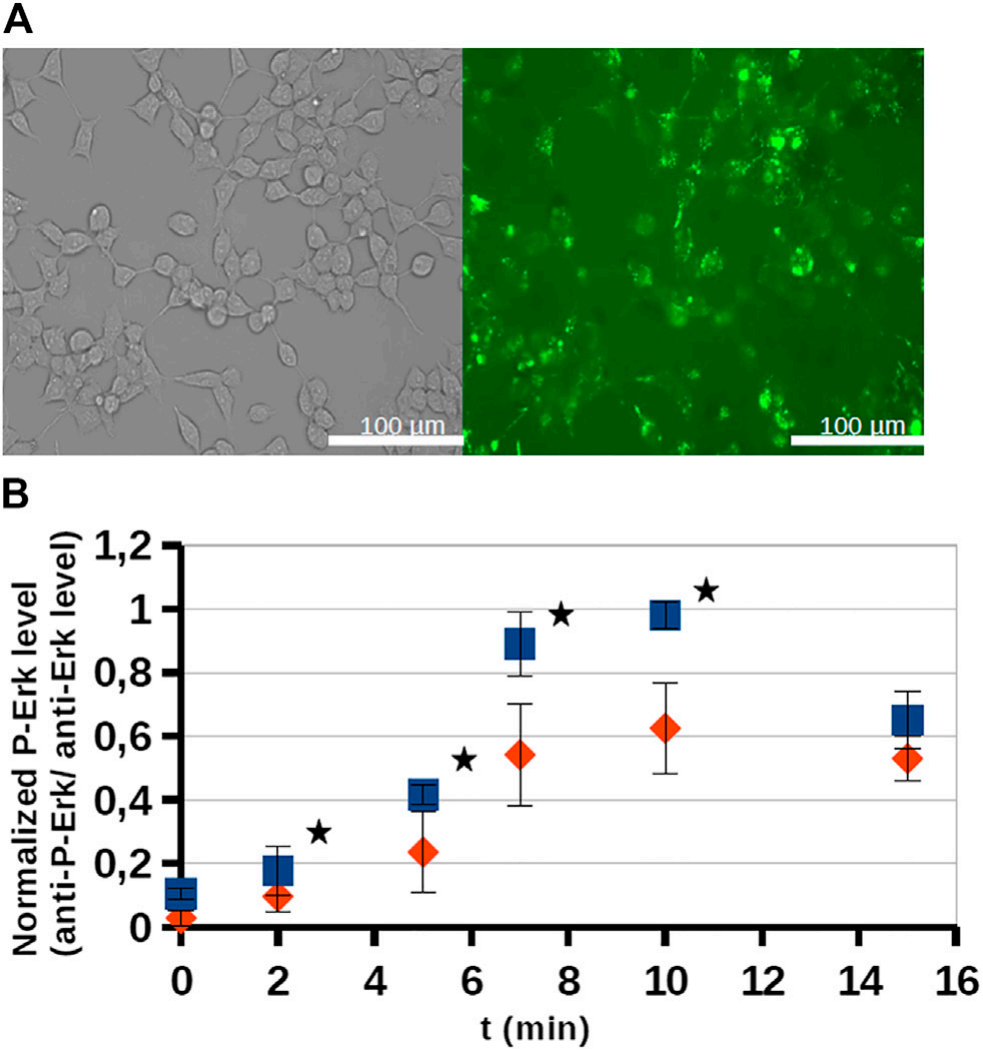
Internalization and intracellular inhibitory activity of an octaarginine conjugate **(3).** The conjugate **(3)** treated cells (20 μM, 5 h) were analyzed by fluorescence microscopy **(A)**, or stimulated by EGF (100 ng/ml). The activity of ERK-pathway was followed by anti-P-ERK antibody using Western-blots **(B)**. The normalized phospho-ERK levels in untreated cells and peptide treated cells are labeled with blue and red, respectively. Error bars show SD calculated based on three independent measurements. * Indicates statistical significance between peptide-treated and control (*p* < 0.05 with two-sided independent Student’s *t*-test).

The effect of an octaarginine conjugate (**3**) on intracellular activity of ERK was studied on HEK-293T cells. First, the internalization of the conjugate was studied by fluorescence microscopy (Figure 4A). It showed internalization after 5 h of treatment at 5 µM concentration.

**FIGURE 5:**
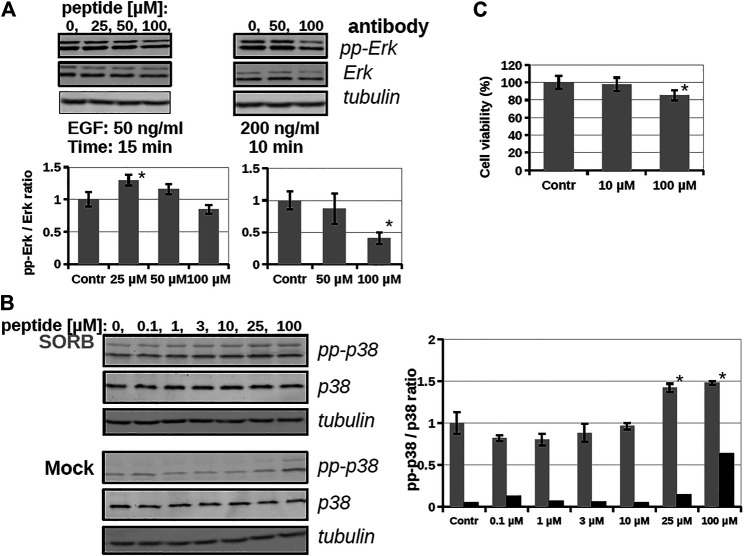
The effect of a bicyclic peptide (16) on ERK and p38 pathways. **(A)** Serum-starved HEKT cells were pre-treated with 0, 25, 50, 100 µM peptide **16** for 2 h, and then stimulated by 50 ng/ml EGF for 15 min or 200 ng/ml EGF for 10 min. Samples were subjected to Western-blot analysis; activity of the ERK pathway was followed by anti-pp-ERK and anti-ERK antibody; anti-tubulin antibody was used as load control and demonstrates equal sample load in addition to the anti-ERK Western-blot signal. Bar charts show the ppERK2/ERK2 intensity ratio of each sample. **(B)** Serum-starved HEKT cells were pre-treated with the peptide **16** at 0.1, 1, 3, 10, 25, 100 µM for 1 h and then stimulated by 0.4 M Sorbitol for 10 min (upper panel) or treated only with the medium (lower panel; Mock). p38 activation was confirmed by using anti-pp-p38 antibody, and the total p38 amount of the samples by using the anti-p38 antibody. The graph shows the pp-p38/p38 intensity ratio of each band obtained from the densitometry of Western-blots in the case of Sorbitol (in grey) or medium (Mock; in black) treatment **(C)** Cell viability test with peptide **16**. HEKT cells were incubated with the peptide at 10 and 100 µM for 24 h, then the viability of cells was determined by PrestoBlue™ reagent according to the manufacturer’s instructions (ThermoFisher Scientific, #A13261). Note that peptide incubation times before stimulation was only up to 2 h at maximun on Panel A and B. Error bars show SD of 3 independent measurements. *Indicates statistical significance between peptide-treated and control (*p* < 0.05 with two-sided independent Student’s *t*-test).

As the conjugate could internalize into cells, its inhibitory effect on intracellular ERK activity was analyzed by using Western-blots (Figure 4B). The cells were stimulated by EGF and the time dependent phosphorylation of ERK1/2 was followed. After EGF stimulation phosphoERK levels normally increase (0–10 min), which at later time points (30–60 min) return to baseline (not shown). When the cells were pre-treated by conjugate **3** (20 μM, 5 h) some reduction was observed in the amount of phosphorylated ERK, meaning that the active form of ERK decreased and indicating that the conjugate attenuated ERK activation. In the case of the most active derivative (bicyclic peptide **16**) HEK-293T cells were pre-treated with the solution of inhibitor peptide (**16**) at 10, 25, 50, and 100 µM concentrations and cells were stimulated with EGF at 50 ng/ml for 15 min (Figure 5A), or with EGF at 200 ng/ml for 10 min. Unexpectedly, peptide **16** increased the phosphorylation of ERK at low concentration (with 30 and 16% at 25 and 50 μM, respectively) and inhibitory activity (15%) was detected only at the highest concentration (100 µM) at 50 ng/ml EGF and 60% at 200 ng/ml EGF. When the inhibition of intracellular p38 was studied the pre-treatment with peptide **16** (0.1, 1, 3, 10, 25, 100 µM) was for 1 h and we applied stimulation with sorbitol for 10 min. The effect of peptide **16** on p38 was somewhat different compared to on ERK1/2 (Figure 5B). At low concentration it inhibited the phosphorylation of p38 (with 18, 20, 12, 4% at 0.1, 1, 3, and 10 μM, respectively), but there was high increase in the phosphorylation of p38 at 25 μM, which was slightly enhanced further at 100 μM, with 42 and 48%, respectively. It is noteworthy, that pre-treatment with these peptides had only marginal effects on cell viability, which, however, was only apparent after long incubation (for 24 h, as shown on Figure 5C).

## Discussion

MAPKs are important components of intracellular signaling pathways and thus they are responsible for many biological process in cells (Cargnello and Roux, 2011). Their aberrant functions are crucial in many pathological symptoms. Development of inhibitors against them, and thus to attenuate their effect in pathological alterations, has turned out to be challenging so far. The most applied strategy, inhibition of kinase activity *via* blocking ATP binding has several limitations. One of them is limited selectivity because of the high homology of ATP binding sites in different families of kinases (Knight and Shokat, 2005). Blocking the interaction of these kinases with their substrates can be a potent strategy to get selective inhibitors. The substrates of MAPKs contain docking motifs to bind to the kinase and this protein–protein interaction results in selectivity in downstream signaling. Many motifs were identified in various kinases and one of them was a sequence from the *N*-terminal part of RHDF1 protein. This peptide—SLQRKKPPWLKLDIPS—showed strong ERK2 and p38 binding (Zeke et al., 2015). This peptide was selected to develop new MAPK based inhibitor(s). In this systematic study our purpose was to develop efficient and cell permeable inhibitor peptide(s) based on the original sequence. As this sequence has good binding ability (K_d_ = 1.8 µM, Table 1) the first approach was to provide them with cell-penetrating tags to improve cellular uptake. These peptides were used successfully to develop cell-permeable MAPK inhibitor peptides (Kelemen et al., 2001; Fu et al., 2008; Gaestel and Kracht, 2009). Octaarginine was selected to deliver the peptide into cells, because it has been successfully used by us to deliver peptides into cells (Bánóczi et al., 2007; Bánóczi et al., 2008a; Világi et al., 2008). The conjugate retained MAPK binding ability and could penetrate into cells and had some inhibitory effect on the ERK pathway (Figure 4). Our hypothesis was that the low cellular stability may reduce the applicability of this construct. Thus, our interest turned toward the optimization of the binding peptide sequence, its cell-penetration and its in-cell stability. First the size of the peptide was reduced. The data suggested that despite that the flanking regions (SLQR and PS) may not act in binding directly based on available structural models of MAPK-docking motif complexes, they have some roles in it (Table 1). A short peptide (**4**) without these flanking regions showed weaker binding. To enhance cellular internalization of peptides, Lys-Arg substitutions were done (**5**) as it was proved that Arg residue(s) has high impact on cell penetration (Mitchell et al., 2000). This modification only slightly reduced binding *in vitro*. Based on these findings extension of both termini with Arg residues was done. While the number of Arg has a lower limit for efficient internalization (Mitchell et al., 2000; Futaki et al., 2001), there is high flexibility in their position (Futaki et al., 2002). The extension of the *C*-terminus with two Arg residues (**6**) induced stronger binding and the addition of an extra Arg to the *C*-terminus (**8**) resulted in the same binding as that of the original sequence. In addition to increased binding these peptides showed increased cellular uptake in HEK-293T cells. Because our latest findings showed that the Dabcyl-group may dramatically increase the internalization of oligoarginines this chromophore group was attached to the *N*-terminal amino group of peptide **8**. Coupling of this group (peptide **10**) slightly attenuated binding, but its fluorescent derivative (peptide **11**) had improved internalization ability into HL-60 and HEK-293T cells (Figure 2D and Figures 3, 4). Its internalization was 10–24 times higher at different concentrations compared to the peptide without the Dabcyl-group. To improve the binding of peptide **10**, several cyclic and bicyclic peptide derivatives were synthesized. There are many examples where cyclic or bicyclic peptides were used as efficient PPI inhibitors (Cardote and Ciulli, 2016; Lennard et al., 2019; Colarusso et al., 2020). First, a medium sized cyclic peptide was made, where the hydrophobic binding region and the spacer sequence (**15**) was cyclized. This cyclic peptide had the same binding as the original peptide, suggesting that cyclization resulted in a structure that is compatible with binding. Next, the same cyclic derivative (**13**) without Ile^14^ was tested. This Ile may promote binding but it is not necessary for it. Our idea was that the cyclization may be enough for binding and the peptide can be shortened. Indeed, this shorter cyclic peptide (**13**) had a slightly better binding. The different size of the cycle may change the structure and the flexibility of the cyclic peptide. When only the region that is responsible for binding to the hydrophobic docking groove was cyclized (**12**) a smaller cyclic peptide was formed, likely having a more rigid structure. When all amino acids were in the cycle (**14**), the structural flexibility was presumably increased. Both cyclic peptides were designed without Ile^14^. As both peptides had decreased binding ability, we could conclude that these cycles do not close the peptide into the proper structure. In the final attempt bicyclic peptides (**16** and **17**) were prepared. These peptides were designed on the basis of the best cyclic form (**13**), but instead of one cycle, the two parts of the peptide—the binding and spacer regions—were incorporated into two different loops. In these constructs the role of Ile^14^ was also examined. Interestingly, the bicyclic peptide with Ile^14^ (**16**) was more effective than the bicyclic peptide without Ile^14^ (**17**). This may suggest that in case of a more rigid structure the extra hydrophobic amino acid (Ile^14^) could promote stronger interaction with the enzyme. It is noteworthy that only the best bicyclic peptide showed stronger binding to ERK2 than to p38. Cyclization can increase the stability of the peptides *in vivo*, suggesting that the bicyclic construct can be stable enough to have inhibitory activity on the intracellular activity of ERK2 or p38. Peptide **16** indeed affected ERK and p38 phosphorylation levels in cells, but our results also showed that it is not straightforward to predict the impact of cell-penetrating docking motif containing peptides on these signaling pathways. There are many interaction partners of these proteins in the cell which can all compete with each other and with the peptides for binding the MAPK docking. Further complexity comes from the fact that some of the MAPK partners are activators (MAPK kinases), others are deactivators (phosphatases) or regulatory proteins (scaffolds) and the relative concentration of all these—in comparison to the “inhibitory” MAPK docking groove targeting peptide‘s intracellular concentration—will collectively determine the final output. For example, Figure 5 shows that peptide **16** at 100 µM has greater inhibitory effect at higher EGF concentration, because the relative concentrations of the interaction partners are different. In the case of p38 pathway activation by sorbitol, the inhibitory effect of peptide **16** was observed at low concentration only, but at higher concentrations the peptide enhanced p38 activation. In addition, the peptide in unstimulated cells (Figure 5, Mock) increased the p38 phosphorylation level, possibly because that peptide interferes with the binding of negative regulators such as phosphatases in the resting state, too. A biological parable that demonstrates the complexity of MAPK docking is the case of the so-called “sevenmaker*”* mutation in the ERK2 MAPK docking groove (D319N). This mutation affects an important docking groove residue and causes elevated ERK2 pathway output in cells because the docking of phosphatases to this mutant is more affected than the binding of upstream activators or downstream substrates (Bott et al., 1994)**.** This “biological” example shows that selective and engineered perturbation of this complex PPI system is conceptually possible but it also suggests that the impact of docking based “inhibitory” peptides on MAPK signaling pathways in cells could be more complex than simple blocking of MAPK phosphorylation. This provides challenges but also opportunities for MAPK activity modulation.

## Data Availability

The original contributions presented in the study are included in the article/Supplementary Material, further inquiries can be directed to the corresponding authors.
