# Supplementary material for: Peptide Based Inhibitors of Protein Binding to the Mitogen-Activated Protein Kinase Docking Groove
Source: Front Mol Biosci. 2021 Jul 1;8:690429. doi: 10.3389/fmolb.2021.690429 (PMC8281026; doi:10.3389/fmolb.2021.690429)
Supplement: Supplementary file 1 [file DataSheet1.docx]

Supplementary Material

# Characterization of compounds by analytical RP-HPLC

Characterization with analytical RP-HPLC was performed on Zorbax SB C18 column (150_4.6mm I.D.) with 5 µm silica (100 Å pore size) column. Linear gradient elution was used: 0 min 0% B; 2 min 0% B; 22 min 90% B with eluent A (0.1% TFA in water) and eluent B (0.1% TFA in acetonitrile-water (80:20, v/v)), flow rate: 1 mL/min, ambient temperature. Peaks were detected at λ= 220 nm.


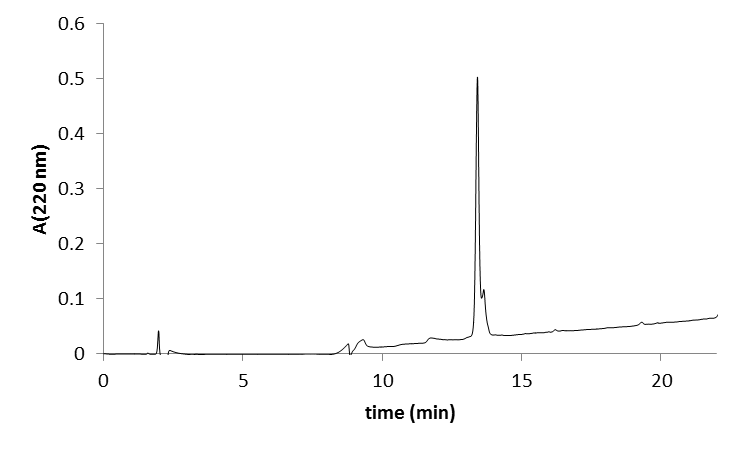


**Supplementary Figure 1.** Analytical RP-HPLC chromatogram of *Cf*-SLQRKKPPWLKLDIPSC(*CH_2_-CO*-RRRRRRRR-*NH_2_*)-*NH_2_*

**Supplementary Figure 2.** Analytical RP-HPLC chromatogram of *H*-SLQRKKPPWLKLDIPSC(*CH_2_-CO*-RRRRRRRR-*NH_2_*)-*NH_2_*


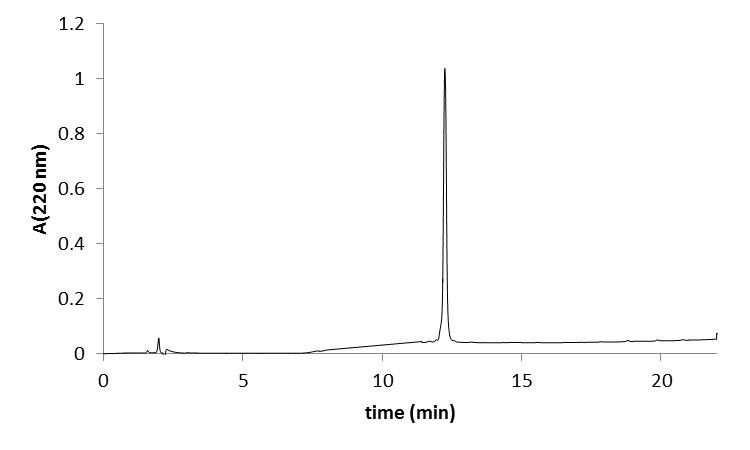


**Supplementary Figure 3.** Analytical RP-HPLC chromatogram of *H*-KKPPWLKLDI-*NH_2_*

**Supplementary Figure 4.** Analytical RP-HPLC chromatogram of *H*-RRPPWLRLDI-*NH_2_*

**Supplementary Figure 5.** Analytical RP-HPLC chromatogram of *H*-RRPPWLRLDIRR-*NH_2_*

**Supplementary Figure 6.** Analytical RP-HPLC chromatogram of *Cf*-RRPPWLRLDIRR-*NH_2_*

**Supplementary Figure 7.** Analytical RP-HPLC chromatogram of *H*-RRRPPWLRLDIRR-*NH_2_*

**Supplementary Figure 8.** Analytical RP-HPLC chromatogram of *Cf*-RRRPPWLRLDIRR-*NH_2_*

**Supplementary Figure 9.** Analytical RP-HPLC chromatogram of *Dabcyl*-RRRPPWLRLDIRRK-*NH_2_*

**Supplementary Figure 10.** Analytical RP-HPLC chromatogram of *Dabcyl*-RRRPPWLRLDIRRK(*Cf*)-*NH_2_*

**Supplementary Figure 11.** Analytical RP-HPLC chromatogram of


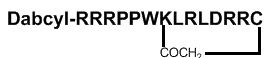


**Supplementary Figure 12.** Analytical RP-HPLC chromatogram of


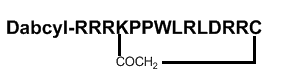


**Supplementary Figure 13.** Analytical RP-HPLC chromatogram of


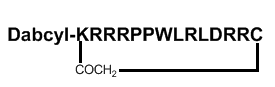


**Supplementary Figure 14.** Analytical RP-HPLC chromatogram of


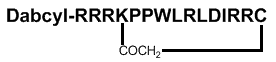


**Supplementary Figure 15.** Analytical RP-HPLC chromatogram of


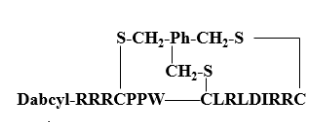


**Supplementary Figure 16.** Analytical RP-HPLC chromatogram of


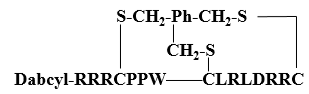


# Characterization of compounds by ESI-MS

The molecular weight of peptides was confirmed by ESI-MS. Bruker Daltonics Esquire 3000 plus (Germany) ion trap mass spectrometer was used. The samples were dissolved in water-acetonitrile solution (50:50) with 0.1% acetic acid. The samples were directly injected with a syringe pump. Parameters: capillary voltage: 4 kV, nebulizer gas: 10 psi, dry gas:4 L/min, heated capillary temperature: 250 ºC.


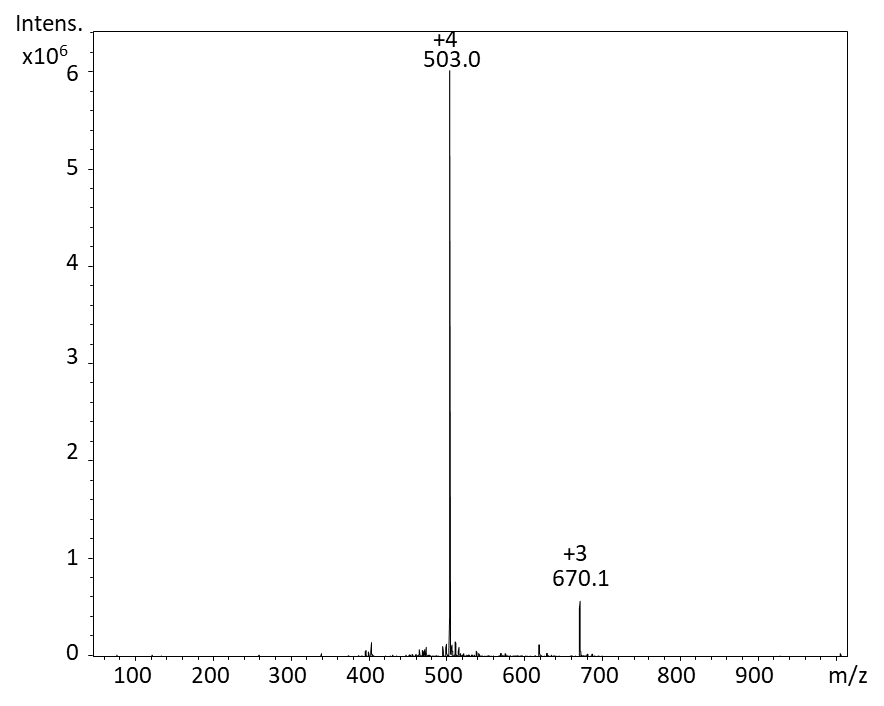


**Supplementary Figure 17.** ESI-MS spectra of *H*-SLQRKKPPWLKLDIPSC-*NH_2_*


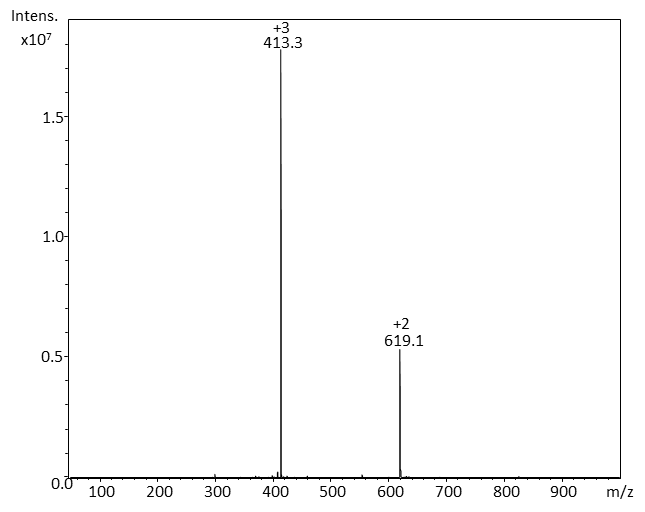


**Supplementary Figure 18.** ESI-MS spectra of *H*-KKPPWLKLDI-*NH_2_*


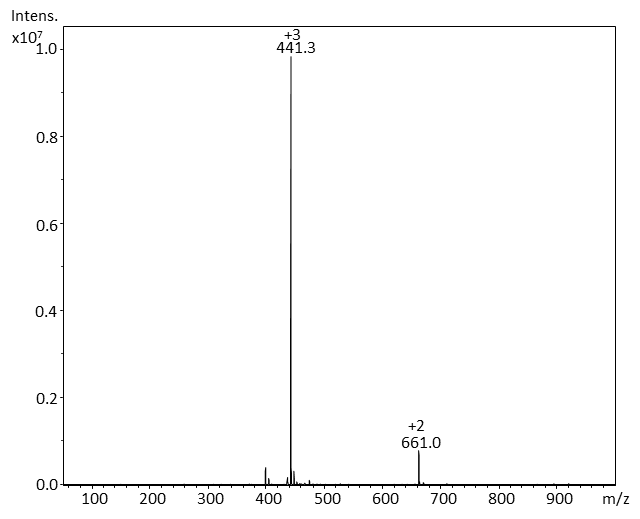


**Supplementary Figure 19.** ESI-MS spectra of *H*-RRPPWLRLDI-*NH_2_*


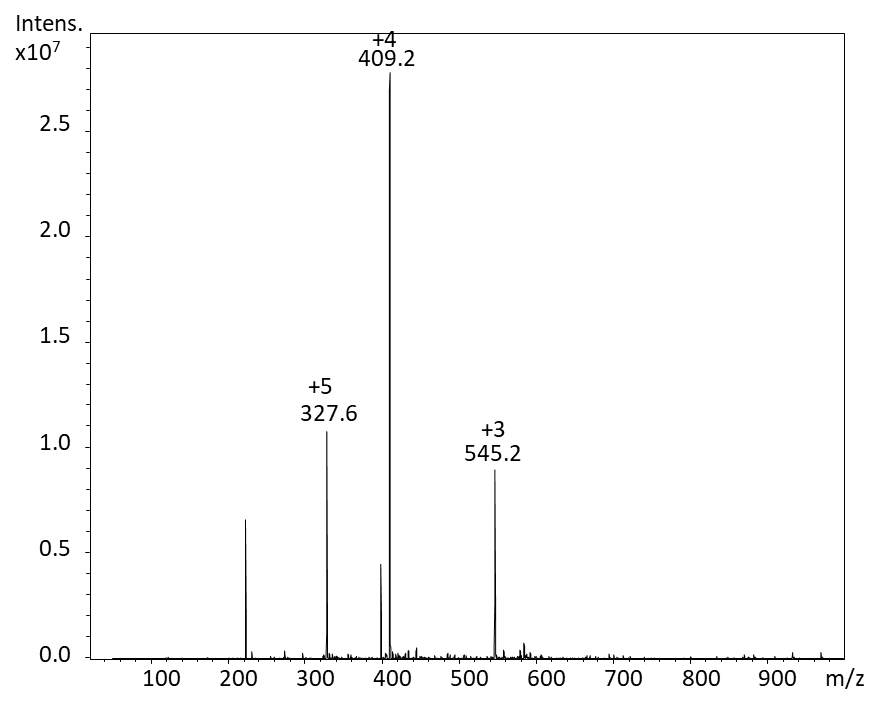


**Supplementary Figure 20.** ESI-MS spectra of *H*-RRPPWLRLDIRR-*NH_2_*


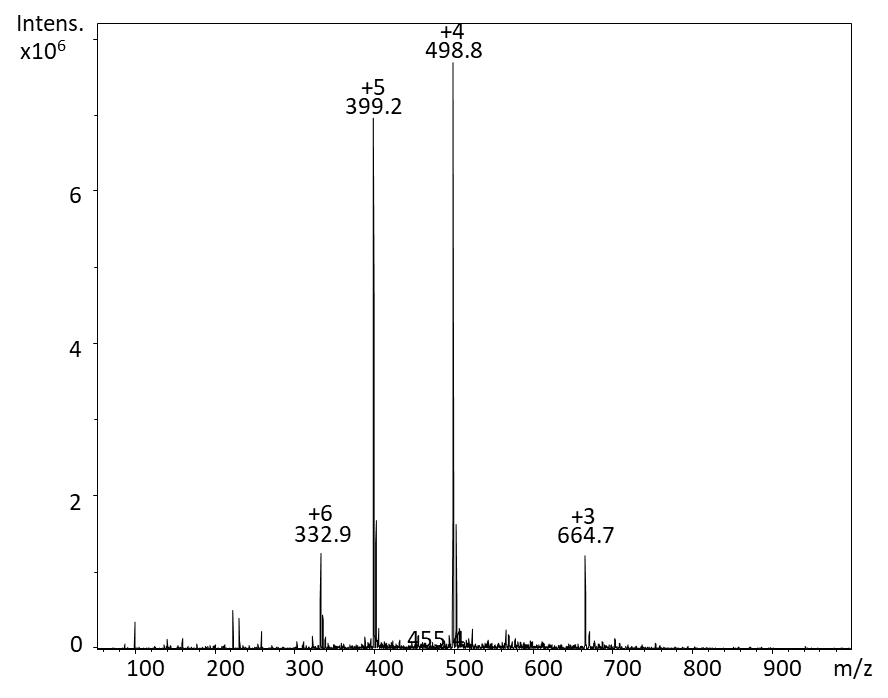


**Supplementary Figure 21.** ESI-MS spectra of *Cf*-RRPPWLRLDIRR-*NH_2_*


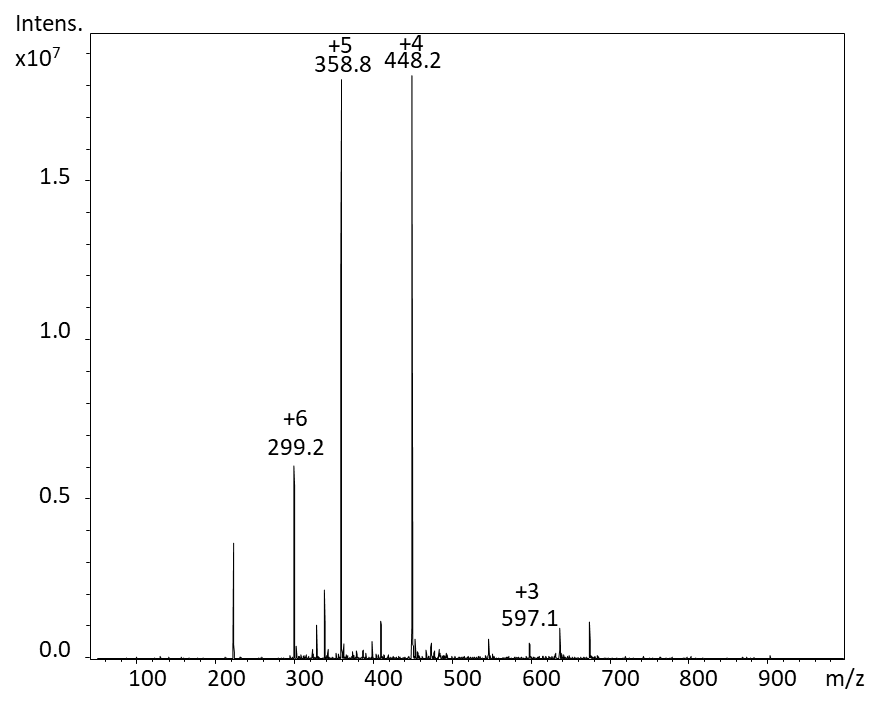


**Supplementary Figure 22.** ESI-MS spectra of *H-*RRRPPWLRLDIRR-*NH_2_*


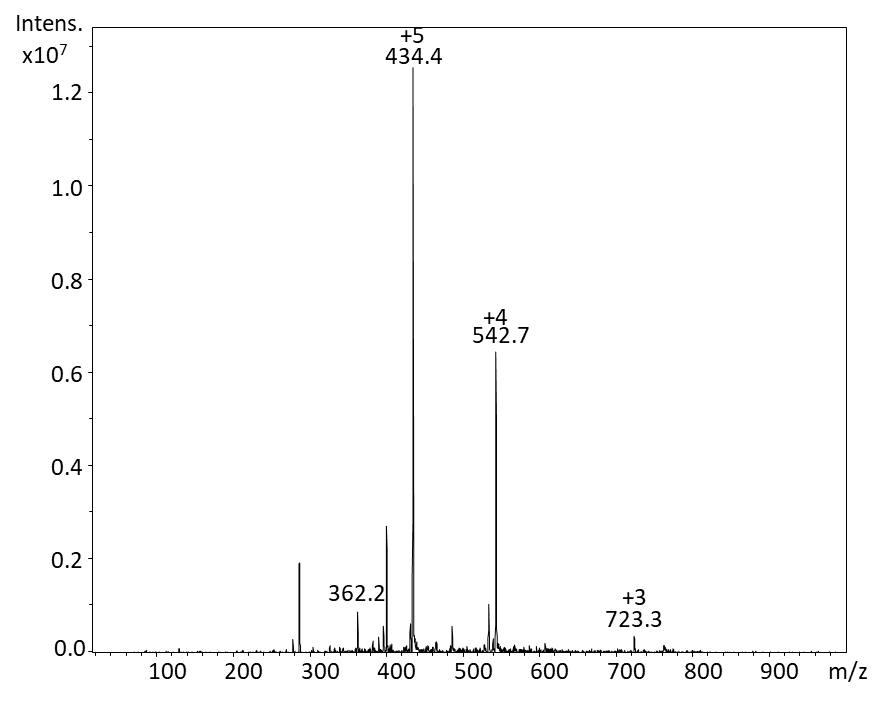


**Supplementary Figure 23.** ESI-MS spectra of *H-*RRRPPWLRLDIRR-*NH_2_*

# Measurement of binding ability of compounds by fluorescence polarization

**Supplementary Figure 24.** **Fluorescence polarization assay for the determination of K_D_ values for ERK2-peptide binding**

**A)** Direct titration of ERK2 with Cf-labeled peptide 1 **B)** Competitive titration with peptide 1 **C)** with peptide 10 and **D)** with peptide 16

**
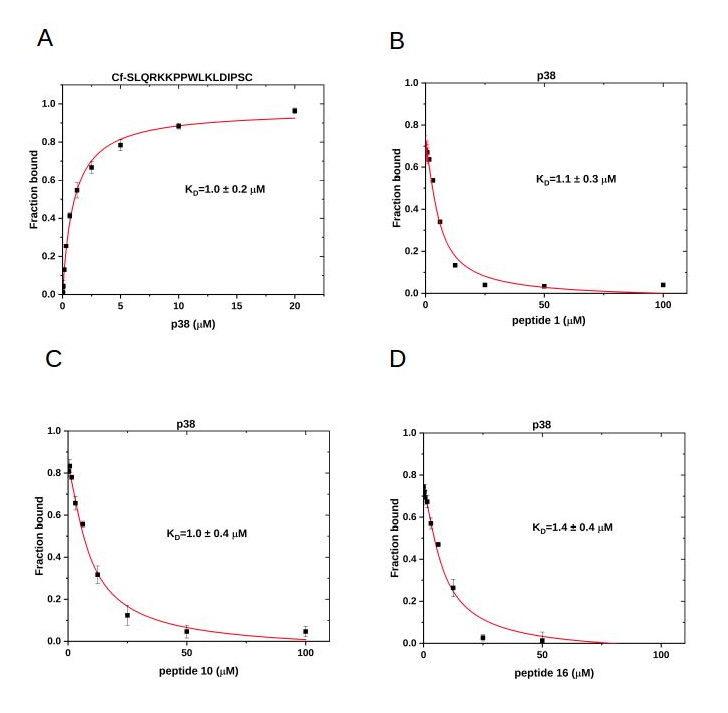
**

**Supplementary Figure 25. Fluorescence polarization assay for the determination of KD values in for p38α-peptide binding**

**A)** Direct titration of p38α with Cf-labeled peptide 1 **B)** Competitive titration with peptide 1 **C)** with peptide 10 and **D)** with peptide 16

# Measurement of EC_50_ value by protein fragments complementation assay

**Supplementary Figure 26.** ***In vitro* activity measurement for the determination of EC_50_ values**

MAPK activity assay was used for the determination of EC_50_ values of the inbibitory peptides. Increasing concentrations of peptide 10 were applied to inhibit pp-ERK2 activity **(A)** or pp-38 activity **(B)** and the initial rate of the reactions were plotted against the logaritm of the peptide concentration (upper graphs).
